# Bilateral lower limb compartment syndrome after intermittent pneumatic compression and elastic stockings: a therapeutic challenge

**DOI:** 10.1590/1677-5449.202301712

**Published:** 2025-08-22

**Authors:** Adenauer Marinho de Oliveira Góes, Clarisse Gripp Aita, Sellena Polyana Soares de Souza Brito, Fernanda Beatriz de Albuquerque Feijó, Matheus Oliveira Feijó, Flávia Beatriz Araujo de Albuquerque

**Affiliations:** 1 Sociedade Brasileira de Angiologia e Cirurgia Vascular SBACV, São Paulo, SP, Brasil.; 2 Hospital Unimed Prime, Belém, PA, Brasil.; 3 Centro Universitário do Estado do Pará CESUPA, Belém, PA, Brasil.; 4 Universidade Federal do Pará UFPA, Belém, PA, Brasil.; 5 Universidade Federal do Paraná UFPR, Hospital das Clínicas HC, Curitiba, PR, Brasil.; 6 Força Aérea Brasileira FAB, Belém, PA, Brasil.; 7 Exército Brasileiro EB, Rio de Janeiro, RJ, Brasil.

**Keywords:** anterior compartment syndrome, fasciotomy, intermittent pneumatic compression devices, postoperative complications, venous thromboembolism, prophylaxis

## Abstract

This article reports the case of a patient who developed bilateral compartment syndrome of the legs after use of intermittent pneumatic compression and compression stockings as prophylactic measures against venous thromboembolism in the perioperative period of plastic surgery. The patient was treated with fasciotomies, followed by uneventful recovery. A literature review is also presented covering diagnosis and treatment of lower limb compartment syndrome and the pathophysiological mechanisms of this clinical condition as a postoperative complication.

## INTRODUCTION

Lower limb (LL) compartment syndrome (CS) is defined as elevated pressure within the myofascial compartment causing compression of neurovascular bundles, provoking intense pain and risk of neurological sequelae, tissue necrosis, and amputation.^1-4^ The most common etiologies include traumatism, especially those involving fractures, burns, popliteal vessel injury, and use of de torniquets. Additionally, the vascular diseases acute arterial occlusion, phlegmasia cerulea dolens and phlegmasia alba dolens can also cause CS.^2,5-7^

The anterior compartment of the leg is the compartment most often involved in CS, containing the tibialis anterior, extensor digitorum longus, extensor hallucis longus, and fibularis tertius muscles, their satellite veins, and the deep fibular nerve.^4,5,8,9^

Pain is the primary clinical manifestation of CS and is characteristically intense and triggered by passive movement. The musculature tends to be edematous and tense. If not treated promptly, CS can cause paresthesia, restricted movement, absent distal pulses, pallor, and cyanosis.^1,7,10-12^

Diagnosis is generally clinical. Differential diagnoses include deep venous thrombosis, popliteal artery entrapment syndrome, tendinitis, myositis, stress fracture, and radiculopathy.^10,12-14^ Supplementary examinations such as magnetic resonance imaging (MRI) and arterial and venous color Doppler ultrasonography (CDUS) are often ordered. However, there are no pathognomonic findings that are specific to CS.^5,9,13^ The gold standard for diagnosis is measurement of compartmental pressure,^10,13^ with values exceeding 30 mmHg generally considered indicative of a need for decompressive fasciotomy.^5,7,10^ However, some authors suggest that this method is insufficiently sensitive and specific, and it has been demonstrated that values greater than 30mmHg can be found without CS being present.^2,3,5,15^

Intermittent pneumatic compression devices (IPCD) and graduated compression elastic stockings (GCES) are often used as mechanical methods of venous thromboembolism (VTE) prevention in both clinical and surgical patients, in conjunction with drug-based prophylaxis, or as an alternative to it when there are formal contraindications to its use.^6,16^ Complications secondary to these methods are rare in clinical practice and the prevalence of CS linked to their use has not been established in the literature.

This article reports a case of LL CS after use of IPCD and GCES for VTE prophylaxis in a patient who had undergone abdominal liposuction surgery. This study follows all of the recommendations set out in resolution 466/2012-CNS, and was analyzed and approved by the Research Ethics Committee (Ethics Appraisal Submission Certificate 80320624.9.0000.5169; Consolidated Opinion number 7014595).

## PART I: CLINICAL SITUATION

A 53-year-old male patient presented at emergency complaining of leg pain. The day before he had undergone abdominal liposuction at a different hospital and IPCD and GCES had been used for VTE prevention. During the immediate postoperative period he had spent 6 hours in the intensive care unit due to systemic arterial hypotension. During the night he had complained of pain in the legs of progressive intensity, and the IPCD and GCES had been removed.

He was discharged with a prescription for analgesics, but after a few hours the pains intensified and he sought care at a different hospital. He had no relevant history of illness. Liposuction had been conducted under peridural anesthesia with sedation and had lasted approximately 2 hours and 30 minutes. GCES and IPCD had been used intraoperatively and postoperatively. Information on use of drug-based VTE prophylaxis was not available and could not be obtained.

On physical examination, the patient was in good general health, with bruising to the flanks compatible with the surgical procedure reported. He denied paresthesia involving the feet and mobility and perfusion were preserved. Palpation identified strong posterior tibial pulses, but pedal pulses were absent. He also felt pain in response to palpation of the anterior compartments of the legs and his calves were discretely edematous, albeit without clubbing or perceptible muscle tension, although assessment was compromised by muscle hypertrophy.

The patient was admitted for analgesia and observation and venous CDUS of the LL was ordered but detected no significant changes.

**Figure 1:**
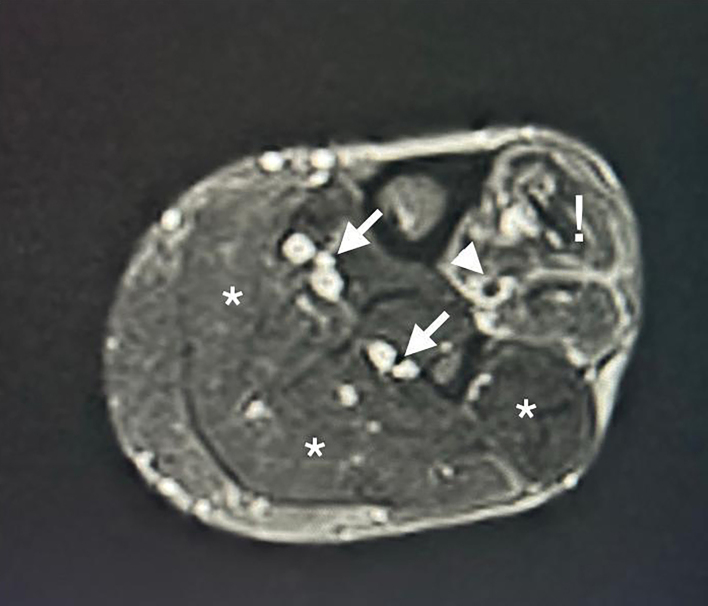
Magnetic resonance imaging of the leg with intravenous contrast, transverse slice: the arrows indicate the posterior tibial and fibular arteries, with flow maintained. The arrowhead indicates the anterior tibial artery, with flow absent. The exclamation mark corresponds to the musculature of the anterior compartment. The asterisks correspond to the musculature of other compartments.

During the night, the pain was unaltered despite analgesia. The following morning, MRI was performed with intravenous contrast, showing muscle edema restricted to the anterior compartments and proximal occlusion of the tibial arteries (Figure 1).

The physical examination findings were unchanged, but the patient mentioned that the night before surgery he had engaged in intensive LL muscle training.

## PART II: WHAT WAS DONE

**Figure 2:**
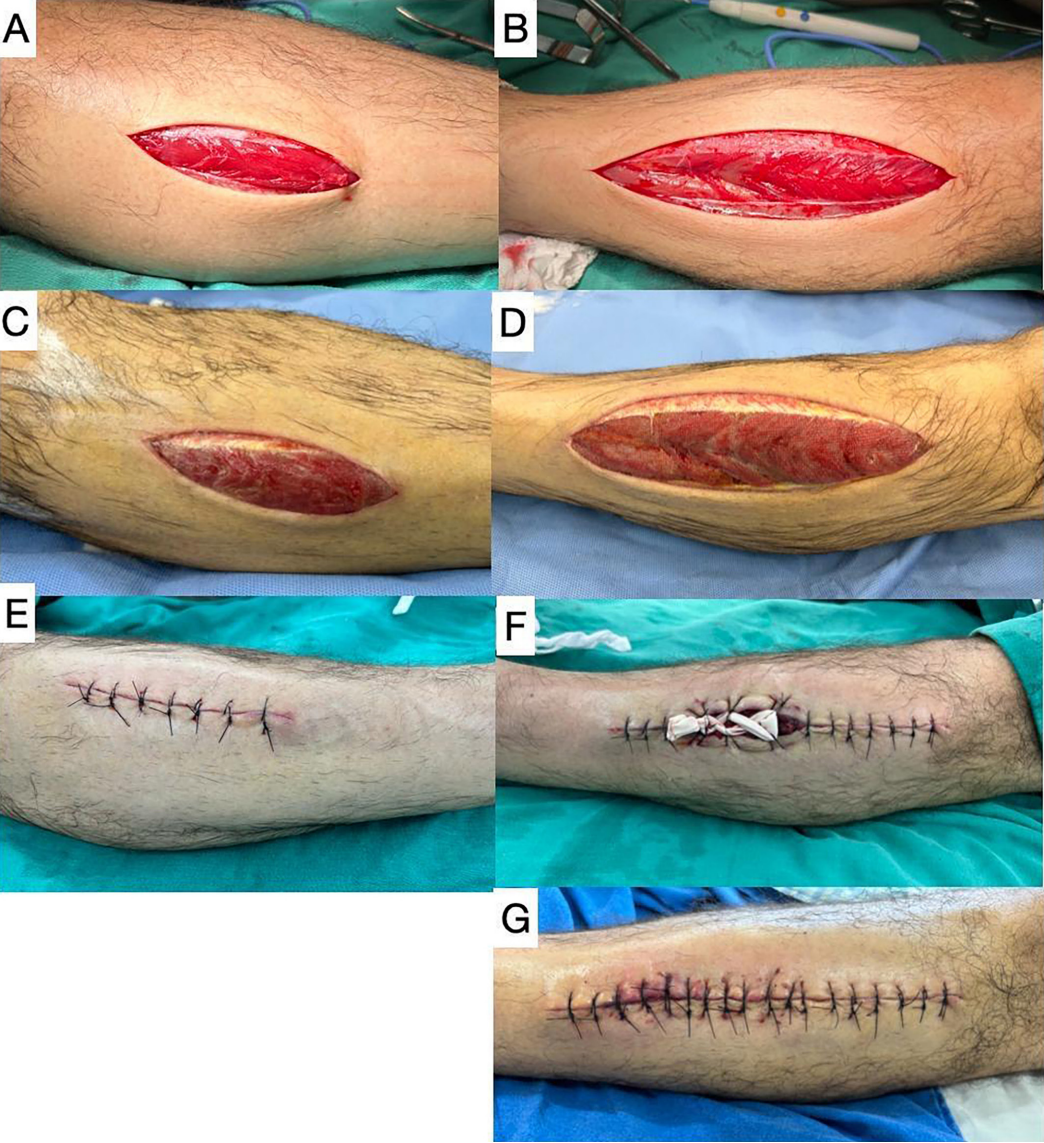
Postoperative course. Images showing the immediate postoperative appearance of the fasciotomies: (A) left leg and (B) right leg. Appearance of the fasciotomies on the sixth postoperative day immediately before and after initial closure: (C) and (E) left leg; (D) and (F) right leg. (G) Final closure of the fasciotomy wound of the right leg.

Pursuing a diagnostic hypothesis of CS, fasciotomies were performed on the anterior compartments of both legs, observing the musculature protruding under tension and immediate reappearance of the pedal pulses, with normal amplitude. On the sixth postoperative day, the left leg fasciotomy wound was closed completely. On the right leg, the extremities of the incision were sutured and approximation of the central portion was achieved with the “shoelace” technique, using a number 4 Penrose drain. On the following day, the wound margins were sufficiently approximated to enable suturing and closure was completed (Figure 2).

**Figure 3:**
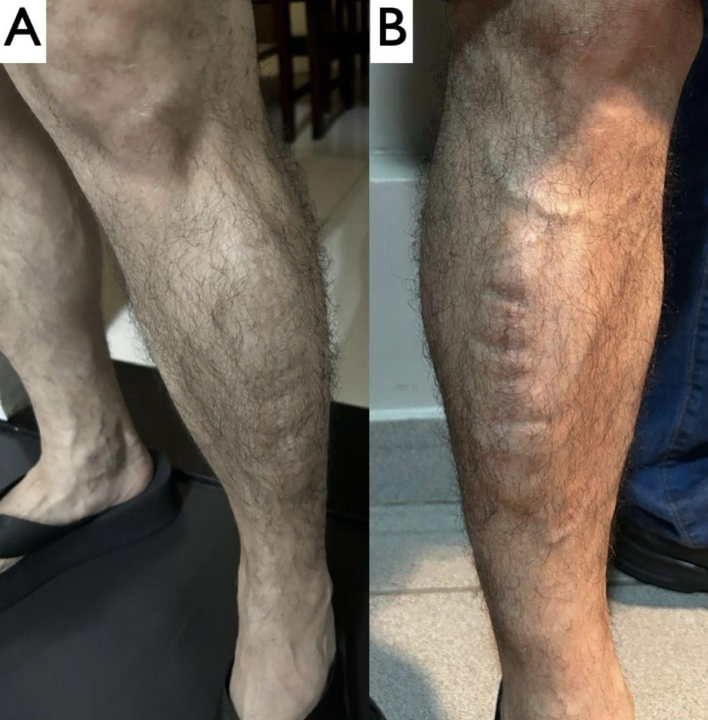
Late postoperative appearance (photographs sent by the patient approximately 2 years after the procedures): (A) left leg and (B) right leg.

The patient was discharged on the third postoperative day following the final procedure. The cutaneous sutures were removed on the 21st postoperative day and the patient was followed-up in outpatients, with no complications, for approximately 30 days. Figure 3 shows photographs sent by the patient, who continued to recover well in the later postoperative period.

## DISCUSSION

Compartment syndrome is a commonplace diagnosis among patients exposed to the classic mechanisms.^2,4,5^ However, in the absence of these mechanisms, and especially in bilateral cases, the diagnosis is undoubtedly less obvious. While there are reports of CS after surgical procedures,^8,9,14,15^ its occurrence is less common. A search of the PubMed/MEDLINE database using the search string “pneumatic compression” AND “compartment syndrome” in title/abstract found fewer than 10 articles published since 1986 describing CS during the postoperative period. Risk factors for CS after surgery include systemic arterial hypotension, as reported by this patient, which causes hypoperfusion of the musculature,^17,18^ and procedures conducted in the lithotomy, Lloyd-Davies (patient in the supine position with flexion of the thighs over the abdomen and the legs over the thighs, both at 90°), and Trendelenburg positions (patient in the supine position with LL elevated at an angle of 15 to 30° in relation to the head)^3, 8,,15^, although it is unclear how these positions affect development of CS. It was not possible to obtain information on the positions in which each patient was operated on, nor their perioperative hemodynamic parameters. Excessive compression due to IPCD malfunction, compounded by the additional compression produced by the GCES, may contribute to development of CS. A patient who is still under the effects of anesthesia may not complain of pain and LL that are covered by drapes during the procedure and by sheets during the postoperative period are often not examined, which delays diagnosis,^9,15^ which itself requires a high degree of clinical suspicion.^2,5^

In the present case, the only complaint was pain, after more than 24 hours since onset, with no paresthesia and maintaining good perfusion of the feet, which is unusual after so many hours. The bilateral absent pedal pulses were not initially considered significant, since this is a common finding in the general population.^16,19^ While manometers that are appropriate for measurement of intercompartmental pressure do exist,^3,7,13^ they are rarely available in practice. For this reason, improvised techniques have been described in which a mercury manometer is connected to a needle.^3,4,7,9^ However, as reported by Tillinghast, fasciotomy is only conducted on the basis of intracompartmental pressure measurements in around 35% of cases.^15^

Factors that limit use of manometry include the absence of standardization of how deep the catheter/needle should be inserted into the muscle compartment, the variability of precision between apparatus, and different recommendations of the exact value of compartmental pressure above which fasciotomy would be indicated.

These limitations have led some authors to recommend that the main purpose of intracompartmental pressure measurement should be to confirm suspicion of CS based on clinical status and that demonstrating elevated pressure in a series of measurements has greater sensitivity and specificity than single measurements for diagnosis of CS.^3,4,13^ In the case reported, compartmental pressure was not measured because an appropriate manometer was not available and because of the limitations described above. Serum markers of muscle necrosis, such as lactate dehydrogenase and creatine phosphokinase, may be elevated after intense exercise and there are reports of CS triggered by exercise among athletes.^1,3,16,20^ However, these markers were not assayed and could have been elevated due to the recent surgical procedure. However, the patient’s LL muscle training the day before surgery, compounded by compression of the calves by the compression devices for VTE prophylaxis, could have contributed to development of CS.

Although there is no specific test for diagnosis of CS, there are methods that facilitate differential diagnosis.^9,13,21^ In the present case, considering the presentation of onset of LL pain in a postoperative patient, venous Doppler was performed to rule out deep venous thrombosis. However, arterial Doppler was not performed because the presence of strong posterior tibial pulses and bilateral absence of pedal pulses were erroneously interpreted as variations of normal anatomy, and not as signs of CS, which was a diagnosis that was not considered initially. It is exactly the possibility of CS with an unusual etiology, as described herein, that is the main motivation for publication of this case report. In this case, MRI detected collapse of the anterior tibial artery and heterogeneity of the muscles of the anterior compartment, suggesting the diagnosis of CS. Moreover, fasciotomies were indicated because of the failure to improve despite rest and analgesia and because the patient reported having engaged in muscle training the day before surgery.

If CS is not treated promptly, neurological complications can occur, the most common if which is “foot drop”, caused by fibular nerve damage, or even amputation secondary to acute arterial ischemia.^1,10^

Treatment is eminently surgical and fasciotomy achieves effective decompression, demonstrated by the protrusion of the swollen musculature, reduced tension and, sometimes, by immediate reappearance of the distal pulses^22^, as was observed in the present case. The time taken for resolution of the edema varies. Smaller fasciotomies can be left open, with healing by secondary intention.^7,23,24^ However, large incisions may need skin grafts, although in some cases, as in the present case, approximation of the cutaneous margins is possible.^4,23,24^ In Portuguese, the shoelace technique has become known as the “*técnica do cadarço*”. There are variations in terms of the elastic material used and the method of fixation, but the technique consists of applying gradual traction, approximating the wound margins and reducing the need for grafts_._^4,7,23,24^

Perioperative VTE is one of the most significant complications of plastic surgery^25^ and mechanical prophylaxis methods, such as GCES and IPCD, are effective for reducing its prevalence.^6,26^ However, the frequency of CS associated with use of these devices is unknown, partly because postoperative complications often go unpublished.

## FINAL COMMENTS

Case reports do not enable definitive conclusions to be drawn, but, based on the literature review, in order to reduce the risk of complications, the following observations can be made: vigorous LL muscle training exercises a short time before surgery should be discouraged; the GCES prescribed should be manufactured specifically for VTE prophylaxis, with maximum compression of approximately 18 mmHg, should be white in color, toeless, and sold in packaging that states they are sterile, and should be chosen to match the circumference of the limb, avoiding excessive compression.

In turn, IPCD should be checked at intervals, to ensure that they are applying intermittent rather than constant compression, for short periods, and at appropriate intensity. The LL should be reassessed frequently, especially in patients unable to report pain, whether because of anesthesia or neurological sequelae. Reduced perfusion, onset of pain, and neurological changes should arouse a suspicion of CS.
