# Supplementary material for: Bilateral lower limb compartment syndrome after intermittent pneumatic compression and elastic stockings: a therapeutic challenge
Source: J Vasc Bras. 2025 Aug 22;24:e20230171. doi: 10.1590/1677-5449.202301712 (PMC12419751; doi:10.1590/1677-5449.202301712)
Supplement: Translation

# Translation: Síndrome compartimental bilateral nos membros inferiores após o uso de compressão pneumática intermitente e meia elástica: um desafio terapêutico

**DOI:** 10.1590/1677-5449.202301711

**Authors:** Adenauer Marinho de Oliveira Góes, Clarisse Gripp Aita, Sellena Polyana Soares de Souza Brito, Fernanda Beatriz de Albuquerque Feijó, Matheus Oliveira Feijó, Flávia Beatriz Araujo de Albuquerque

**Affiliations:** 1 Sociedade Brasileira de Angiologia e Cirurgia Vascular SBACV, São Paulo, SP, Brasil.; 2 Hospital Unimed Prime, Belém, PA, Brasil.; 3 Centro Universitário do Estado do Pará CESUPA, Belém, PA, Brasil.; 4 Universidade Federal do Pará UFPA, Belém, PA, Brasil.; 5 Universidade Federal do Paraná UFPR, Hospital das Clínicas HC, Curitiba, PR, Brasil.; 6 Força Aérea Brasileira FAB, Belém, PA, Brasil.; 7 Exército Brasileiro EB, Rio de Janeiro, RJ, Brasil.

**Keywords:** síndrome do compartimento anterior, fasciotomia, dispositivos de compressão pneumática intermitente, complicações pós-operatórias, tromboembolismo venoso, profilaxia

## Abstract

No presente estudo, descreve-se o caso de um paciente que desenvolveu síndrome compartimental nos membros inferiores após o uso de dispositivo de compressão pneumática intermitente e meias elásticas como método de profilaxia para tromboembolismo venoso no perioperatório de cirurgia plástica. O paciente foi tratado com fasciotomias e evoluiu sem intercorrências. Ademais, apresenta-se uma revisão da literatura sobre o diagnóstico e o tratamento da síndrome compartimental dos membros inferiores, assim como os mecanismos fisiopatológicos desta condição clínica como complicação pós-operatória.

## INTRODUÇÃO

A síndrome compartimental (SC) dos membros inferiores (MMII) é definida pelo aumento da pressão no compartimento miofascial que leva a compressão dos feixes neurovasculares, provocando dor intensa, risco de sequelas neurológicas, necrose tecidual e amputação^1-4^. As principais etiologias incluem traumatismos, especialmente os associados a fraturas, queimaduras, lesões dos vasos poplíteos e uso de torniquete. Entre as doenças vasculares, a oclusão arterial aguda e as flegmasias cerúlea e alba *dolens* também podem acarretar a SC^2,5-7^.

O compartimento anterior da perna, que contém os músculos tibial anterior, fibular lateral, tibiais posteriores superficial e profundo, a artéria tibial anterior, suas veias satélites e o nervo fibular profundo, é o mais frequentemente acometido na SC^4,5,8,9^.

A principal manifestação clínica da SC é a dor, caracteristicamente intensa e desencadeada por movimentação passiva. A musculatura costuma estar edemaciada e tensa. Quando não tratada precocemente, a SC pode levar à parestesia, restrição à movimentação, ausência de pulsos distais, palidez e cianose^1,7,10-12^.

O diagnóstico frequentemente é clínico. Diagnósticos diferenciais incluem trombose venosa profunda, síndrome do aprisionamento da artéria poplítea, tendinite, miosite, fratura por estresse e radiculopatia^10,12-14^. Exames complementares como a ressonância nuclear magnética (RNM) e o eco-Doppler colorido (EDC) arterial e venoso são frequentemente realizados. No entanto, não há achados patognomônicos ou específicos para a SC^5,9,13^. O padrão-ouro para o seu diagnóstico é a aferição da pressão compartimental^10,13^, sendo que valores acima de 30 mmHg costumam indicar a necessidade de fasciotomia descompressiva^5,7,10^. Entretanto, alguns autores sugerem que esse método não é sensível e específico o suficiente, e já foi demonstrado que valores acima de 30mmHg podem ocorrer sem a concomitância de SC^2,3,5,15^.

Dispositivos de compressão pneumática intermitente (DCPIs) e meias elásticas de compressão graduada (MECGs) são frequentemente utilizados como métodos de prevenção mecânica de tromboembolismo venoso (TEV) em pacientes clínicos e cirúrgicos, em associação à farmacoprofilaxia ou como alternativa a esta, quando houver contraindicação formal ao seu uso^6,16^. As complicações desses métodos não são frequentes na prática clínica, e a prevalência de SC associada ao seu uso não está estabelecida na literatura.

Neste artigo, relata-se um caso de SC dos MMII após utilização de DCPI e MECGs na profilaxia do TEV em paciente submetido a uma cirurgia de lipoaspiração abdominal. O estudo segue todas as recomendações preconizadas pela resolução 466/2012-CNS, e foi avaliado e aprovado pelo Comitê de Ética em Pesquisa (Certificado de Apresentação de Apreciação Ética 80320624.9.0000.5169; número do Parecer Consubstancial 7014595).

## PARTE I: SITUAÇÃO CLÍNICA

Um paciente do sexo masculino, de 53 anos de idade, foi atendido no setor de emergência com queixa de dor intensa nas pernas. No dia anterior, havia sido submetido a uma lipoaspiração abdominal em outro hospital e fez uso de DCPI e MECGs para prevenção de TEV. No pós-operatório imediato, ficou 6 horas em unidade de terapia intensiva devido à hipotensão arterial sistêmica. Durante a noite, relatou surgimento de dores nas pernas, de intensidade progressiva, e o DCPI e as MECGs foram removidos.

Recebeu alta com prescrição de analgesia, porém, após poucas horas, as dores se intensificaram, o que o fez procurar atendimento em outro hospital. O paciente não tinha antecedentes mórbidos relevantes. A cirurgia foi realizada sob anestesia peridural e sedação, com duração aproximada de 2 horas e 30 minutos. MECGs e DCPI foram usados no intra e pós-operatório. Não foi possível obter informações sobre a utilização de farmacoprofilaxia para TEV.

À ectoscopia, apresentava bom estado geral e hematomas em flancos, compatíveis com o procedimento cirúrgico relatado. Negava parestesia nos pés e, ao exame físico, apresentava motricidade e perfusão preservadas. Na palpação, os pulsos tibiais posteriores eram amplos, porém, os pulsos pediosos estavam ausentes. Além disso, sentia dor à palpação dos compartimentos anteriores das pernas, e as panturrilhas encontravam-se discretamente edemaciadas, sem empastamento ou tensão muscular perceptível, embora a avaliação estivesse prejudicada pela hipertrofia da musculatura.

O paciente foi internado para analgesia e observação, e foi solicitado um EDC venoso de MMII, o qual não detectou alterações significativas.

Durante a noite, a dor permaneceu inalterada, apesar da analgesia. Na manhã seguinte, foi solicitada uma RNM com contraste endovenoso, que demonstrou edema muscular restrito aos compartimentos anteriores e oclusão proximal de artérias tibiais anteriores (Figura 1).

**Figura 1:** Ressonância nuclear magnética de perna com contraste endovenoso, corte transversal: as setas apontam as artérias tibial posterior e fibular, com fluxo preservado. A ponta de seta indica a artéria tibial anterior, sem fluxo. O ponto de exclamação corresponde à musculatura do compartimento anterior. Os asteriscos correspondem à musculatura dos outros compartimentos.

Quanto ao exame físico, não houve mudanças. Entretanto, o paciente referiu que, na véspera da cirurgia, havia realizado musculação intensa de MMII.

## PARTE II: O QUE FOI FEITO

Com a hipótese diagnóstica de SC, foram realizadas fasciotomias de compartimentos anteriores de pernas, observando-se protrusão da musculatura sob tensão e reaparecimento imediato de pulsos pediosos, com amplitude normal. No sexto dia de pós-operatório, realizou-se a síntese completa da fasciotomia na perna esquerda. Na perna direita, as extremidades da incisão foram suturadas, e a porção central foi aproximada com a técnica do “cadarço”, utilizando-se um dreno de Penrose número 4. No dia seguinte, as bordas cutâneas estavam aproximadas e a síntese foi completada (Figura 2).

**Figura 2:** Evolução pós-operatória. Aspecto no pós-operatório imediato das fasciotomias: (A) perna esquerda e (B) direita. Aspecto das fasciotomias no sexto dia pós-operatório imediatamente antes e após a síntese: (C) e (E) perna esquerda; (D) e (F) perna direita. (G) Síntese complementar da fasciotomia da perna direita.

O paciente recebeu alta no terceiro dia de pós-operatório do último procedimento. Os pontos cutâneos foram retirados no 21º dia de pós-operatório e o paciente foi acompanhado ambulatorialmente, sem intercorrências, por aproximadamente 30 dias. A Figura 3 apresenta fotografias enviadas pelo paciente, que manteve boa evolução no pós-operatório tardio.

**Figura 3:** Aspecto no pós-operatório tardio (fotografias enviadas pelo paciente após aproximadamente 2 anos de pós-operatório): (A) perna esquerda e (B) perna direita.

## DISCUSSÃO

O diagnóstico de SC em pacientes expostos a mecanismos clássicos é comumente realizado^2,4,5^. Entretanto, a ausência desses mecanismos, especialmente em casos bilaterais, certamente torna o diagnóstico menos óbvio. Embora haja relatos de SC após procedimentos cirúrgicos^8,9,14,15^, sua ocorrência é infrequente. Uma busca na base de dados PubMed/MEDLINE, utilizando a estratégia “pneumatic compression” AND “compartment syndrome” no título/resumo, revelou menos de 10 artigos publicados desde 1986 relatando SC no pós-operatório. Entre os fatores de risco após uma cirurgia estão a hipotensão arterial sistêmica, como referida pelo paciente, levando à hipoperfusão muscular^17,18^, e os procedimentos realizados nas posições de litotomia, Lloyd-Davies (paciente em decúbito dorsal com flexão das coxas sobre o abdome e das pernas sobre as coxas, ambas em 90°) e Trendelenburg (paciente em decúbito dorsal com elevação dos MMII em uma angulação de 15 a 30° em relação à cabeça)^3,8,15^, embora não esteja claro como essas posições influenciam o desenvolvimento da SC. Não foi possível obter a informação sobre a posição na qual o paciente foi operado, nem os parâmetros hemodinâmicos perioperatórios. Compressão excessiva por mau funcionamento do DCPI, associada à compressão adicional pelas MECGs, pode contribuir para o surgimento de SC. Se o paciente ainda estiver sob anestesia, pode não referir dor e, por estarem cobertos pelos campos cirúrgicos no intraoperatório ou por lençóis no pós-operatório, os MMII frequentemente não são avaliados, o que retarda o diagnóstico^9,15^, que exige elevado grau de suspeição^2,5^.

Neste caso, a única queixa era dor, com evolução do quadro há mais de 24 horas, sem parestesia e mantendo boa perfusão dos pés, o que não é comum após tantas horas. Inicialmente, a ausência bilateral de pulso pedioso não foi valorizada, pois é uma ocorrência frequente na população geral^16,19^. Embora existam manômetros apropriados para a aferição da pressão intracompartimental^3,7,13^, eles raramente estão disponíveis. Por isso, já foram descritas improvisações, adaptando-se medidores de esfigmomanômetros a agulhas^3,4,7,9^. Entretanto, como relatado por Tillinghast, a realização de fasciotomias mediante aferição da pressão intracompartimental é realizada em apenas cerca de 35% dos casos^15^.

Entre os fatores que limitam o uso da manometria, estão a falta de padronização na profundidade em que o cateter/agulha deve ser inserido no compartimento muscular, a variabilidade de precisão entre os equipamentos e a divergência quanto ao valor exato da pressão compartimental acima do qual estaria indicada a realização da fasciotomia.

Diante dessas limitações, há autores que afirmam que a aferição da pressão intracompartimental tem como principal função ratificar a suspeita de SC baseada no quadro clínico, e que a demonstração de pressões elevadas em medidas seriadas apresenta maior sensibilidade e especificidade do que medidas isoladas para o diagnóstico da SC^3,4,13^. No caso relatado, a mensuração da pressão compartimental não foi realizada devido à indisponibilidade do manômetro apropriado e às limitações acima descritas. Elevações de marcadores séricos de necrose muscular, como desidrogenase lática e creatinofosfoquinase, podem ocorrer após exercícios intensos, e há relatos de SC desencadeada por exercício em atletas^1,3,16,20^. Entretanto, esses marcadores não foram dosados e poderiam estar elevados devido ao procedimento cirúrgico recente. Contudo, a musculação de MMII na véspera da cirurgia, associada à compressão das panturrilhas pelos dispositivos compressivos de profilaxia de TEV, pode ter contribuído para o desenvolvimento de SC.

Apesar de não haver um exame complementar específico para diagnóstico da SC, existem métodos que auxiliam no diagnóstico diferencial^9,13,21^. No caso relatado, considerando-se o surgimento de dor nos MMII em um paciente em pós-operatório, um Doppler venoso foi executado para descartar trombose venosa profunda. No entanto, o Doppler arterial não foi realizado, já que a presença de pulsos tibiais posteriores amplos e a ausência bilateral do pulso pedioso foram equivocamente interpretadas como variações da anatomia normal, e não como sinais de SC, diagnóstico que não foi aventado inicialmente. A possibilidade de SC diante de uma etiologia não habitual, como a aqui relatada, é justamente a principal motivação desta publicação. Neste caso, a RNM, ao detectar colabamento da artéria tibial anterior e heterogeneidade dos músculos do compartimento anterior, sugeriu o diagnóstico. Além disso, as fasciotomias foram indicadas pela ausência de melhora, apesar do repouso e da analgesia, e pelo relato de musculação na véspera da cirurgia.

Caso a SC não seja tratada precocemente, podem ocorrer complicações neurológicas, sendo a mais frequente o “pé caído”, devido à lesão do nervo fibular, ou mesmo evolução para amputação secundária à isquemia arterial aguda^1,10^.

O tratamento é eminentemente cirúrgico, com fasciotomias promovendo descompressão efetiva, constatada pela protrusão da musculatura, diminuição da tensão e, ocasionalmente, pelo reaparecimento imediato de pulsos distais^22^, como observado neste caso. A resolução do edema ocorre em período variável. Fasciotomias pequenas podem ser deixadas abertas, com cicatrização por segunda intenção^7,23,24^. No entanto, grandes incisões podem requerer enxerto de pele e, em alguns casos, assim como o aqui relatado, a reaproximação das bordas cutâneas é possível^4,23,24^. A técnica de *shoelace* foi difundida em português como técnica do “cadarço”. Existem variações quanto ao material elástico e a forma de ancorá-lo, mas a técnica consiste na tração gradativa, aproximando as bordas da ferida e diminuindo a necessidade de enxertos^4,7,23,24^_._

O TEV perioperatório é uma das complicações mais significativas da cirurgia plástica^25^, e os métodos de profilaxia mecânica, como as MECGs e os DCPIs, são eficazes em diminuir sua prevalência^6,26^. Entretanto, a frequência de SC associada ao uso desses dispositivos não é conhecida, em parte porque complicações pós-operatórias frequentemente não são publicadas.

## CONSIDERAÇÕES FINAIS

Relatos de caso não permitem conclusões definitivas, porém, com base na revisão da literatura, a fim de reduzir os riscos de complicações, as seguintes observações podem ser feitas: exercícios vigorosos de musculação dos MMII, pouco tempo antes de cirurgias, devem ser desaconselhados; as MECGs prescritas devem ser aquelas fabricadas especificamente para profilaxia de TEV, com compressão máxima de aproximadamente 18 mmHg, de cor branca, sem ponteira, comercializadas em embalagens que alertam que o conteúdo é estéril, e devem ser escolhidas de acordo com a circunferência do membro, evitando compressão excessiva.

Os DCPIs devem ser periodicamente verificados, a fim de garantir que promovam compressão intermitente, não constante, por períodos curtos e com intensidade adequada. Os MMII devem ser frequentemente reavaliados, sobretudo nos pacientes incapazes de referir dor, seja por anestesia ou sequelas neurológicas. Diminuição da perfusão, surgimento de dor e alterações neurológicas devem levar à suspeita de SC.
